# Obesity and Preventive Intervention Among Children: A Narrative Review

**DOI:** 10.7759/cureus.54520

**Published:** 2024-02-20

**Authors:** Sayali Umekar, Abhishek Joshi

**Affiliations:** 1 School of Epidermology and Public Health, Datta Meghe Institute of Higher Education and Research, Wardha, IND; 2 Community Medicine, Datta Meghe Institute of Higher Education and Research, Wardha, IND

**Keywords:** unhealthy diet, sedentary behaviors, physical activity, overweight, childhood obesity

## Abstract

Childhood obesity has become a major public health concern around the world, with a rise in prevalence over the last few decades. This abstract provides an overview of pediatric obesity, including its causes, implications, and potential treatments. Childhood obesity is caused by a complex combination of environmental, genetic, and behavioral variables. A child's likelihood of developing obesity is influenced by factors, such as socioeconomic status, family dynamics, and cultural norms. Childhood obesity leads to extensive repercussions, elevating the risk of chronic conditions, such as diabetes, cardiovascular diseases, and mental health challenges. Furthermore, children dealing with obesity often face social stigmatization, diminished self-esteem, and academic struggles. Efforts to prevent and manage childhood obesity should employ a comprehensive and multi-tiered approach. This involves enacting policies geared toward enhancing nutrition in schools and communities, advocating for increased physical activity (PA), and curbing sedentary behaviors.

## Introduction and background

Obesity is derived from the Latin word “obesus," which means pump or having eaten oneself's fat [1]. Furthermore, obesity is caused by an imbalance or disparity in calorie intake and energy expenditure. Childhood obesity refers to an unhealthy excess of body fat that can adversely affect a child's health. The increasing worldwide occurrence of obesity seems largely influenced by environmental factors, lifestyle decisions, and cultural contexts [2]. Obesity is a complex, multifactorial disease [3]. Childhood obesity has emerged as a global health challenge due to its heightened occurrence across both developed and developing nations [4]. Currently, approximately 170 million children worldwide are overweight or obese [4,5]. About 340 million children and teenagers, ages five to 19, were thought to be overweight or obese in 2016.

By 2020, almost 39 million children under the age of five were overweight or obese [6]. Obesity is a serious global health concern since it is linked to a higher chance of dying early and the appearance of numerous health problems in later life, such as cancer, diabetes, cardiovascular disease, and several other mental and physical conditions [7]. Childhood obesity serves as a significant contributing factor to the development of several chronic conditions associated with diet, including late-life conditions, such as heart disease, high blood pressure, stroke, type II diabetes, and several types of cancer [8]. It also has negative psychological effects, such as anxiety, sadness, sleep difficulties, and low self-esteem, which have an impact on children's social and educational interactions [7]. A number of variables, such as less outdoor physical activity (PA), more time spent watching television and using screens, living in urban areas, and coming from wealthy families, contribute to the growing incidence of childhood obesity and overweight in India [9]. Medical conditions, such as type II diabetes, hypertension, and hypercholesterolemia, traditionally prevalent in adults, are increasingly emerging among younger populations [10].

Overweight and obesity are caused by poor eating habits, including the consumption of cold beverages and fast food, skipping meals, insufficient intake of fruits and vegetables, and frequent dining out. The current World Health Organization (WHO) guidelines state that children and teenagers between the ages of five and 17 should participate in 60 minutes a day of moderate-to-vigorous physical activity [11]. Unhealthy eating habits, a sedentary lifestyle marked by irregular sleep schedules, prolonged screen time, and inadequate physical activity are all factors that have a direct impact on children's body weight [8]. Physical activity and a healthy diet are the fundamentals of obesity control and prevention [12]. Physical activity is required for children's normal growth and development.

Body mass index (BMI) is a commonly used tool to assess weight in relation to height, providing a simple measure to categorize overweight and obesity among adults. It is calculated by dividing the weight of a person in kilograms by the square of their height in meters (kg/m^2^) [6]. A BMI of 85-94 is categorized as "overweight," whereas a BMI of 95 is classified as "obese" depending on age and gender, based on the Centers for Disease Control and Prevention (CDC, 2018) [6].

## Review

Methodology

**Figure 1:**
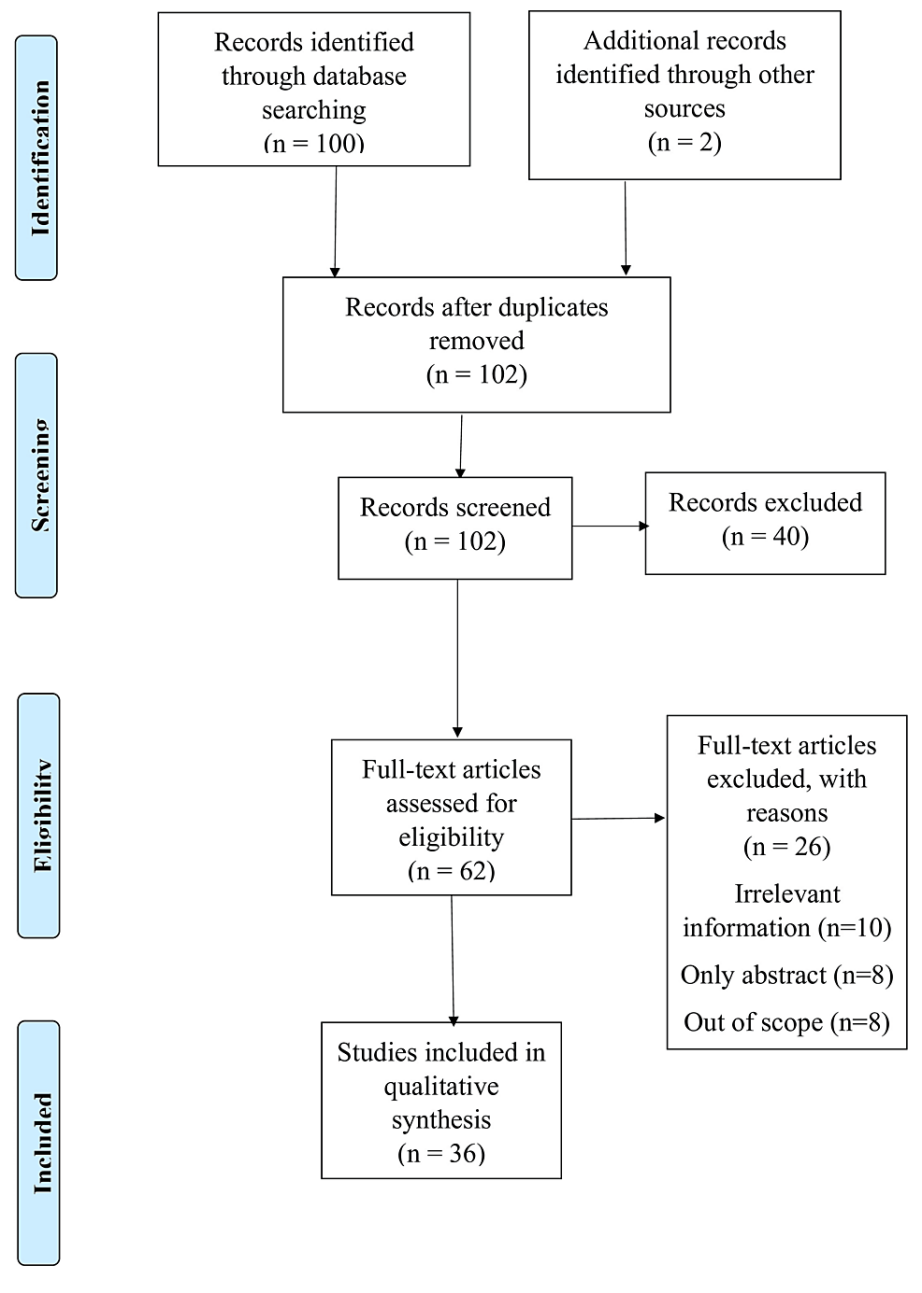
Selection process of articles used in this study. Adopted from the Preferred Reporting Items for Systematic Reviews and Meta-Analyses (PRISMA).

We searched PubMed and Google Scholar using the terms "childhood obesity" and "overweight." We also used the keywords "childhood obesity," "overweight," "physical activity," "sedentary behaviors," and "unhealthy diet." The study language is English, and studies that were in other languages were excluded. In addition, filters were applied, such as full text, during the process of review (Figure 1).

Discussion

Obesity is described as abnormal or excessive fat accumulation, which can affect health. A substantial amount of research indicates that creating and maintaining healthy routines at home can be a useful strategy for preventing obesity [13,14]. Obesity is increasing at a quicker rate in adolescents than in adults [15,16]. Obesogenic habits can have a long-term effect on an adult's weight status and health, often starting in childhood [13,15]. In children, inadequate dietary habits contribute to the rise in obesity, with parents and caregivers exerting the most significant influence on shaping children’s eating behaviors [15,17]. Since 1975, global obesity has nearly tripled. As of 2017, data from the WHO's Global Health Observatory reveals that there are more than 340 million children and adolescents between the ages of five and 19 who are grappling with obesity worldwide [15]. The number of overweight or obese children under five in 2020 was 39 million [6].

Obesity is associated with several health problems, such as depression, type II diabetes, hypertension, cardiovascular disease (CVD), and respiratory problems [13,18]. Physical inactivity is one of the leading causes of obesity-related comorbid disorders [13]. Because the majority of children and adolescents are physically inactive, which puts them at risk for high obesity rates and related diseases like CVD, the promotion of PA programs has grown in importance within health policy [15,19]. Obesity plays a crucial role as a preventable risk factor and a disease influencer in various respiratory conditions, contributing significantly to symptoms like wheezing, difficulty breathing, and breathlessness in both adults and children. Its effects are seen in the frequency and seriousness of various lung conditions, as was most prominently demonstrated during the SARS-CoV-2 pandemic [20-22].

Obesity Around the World

**Figure 2:**
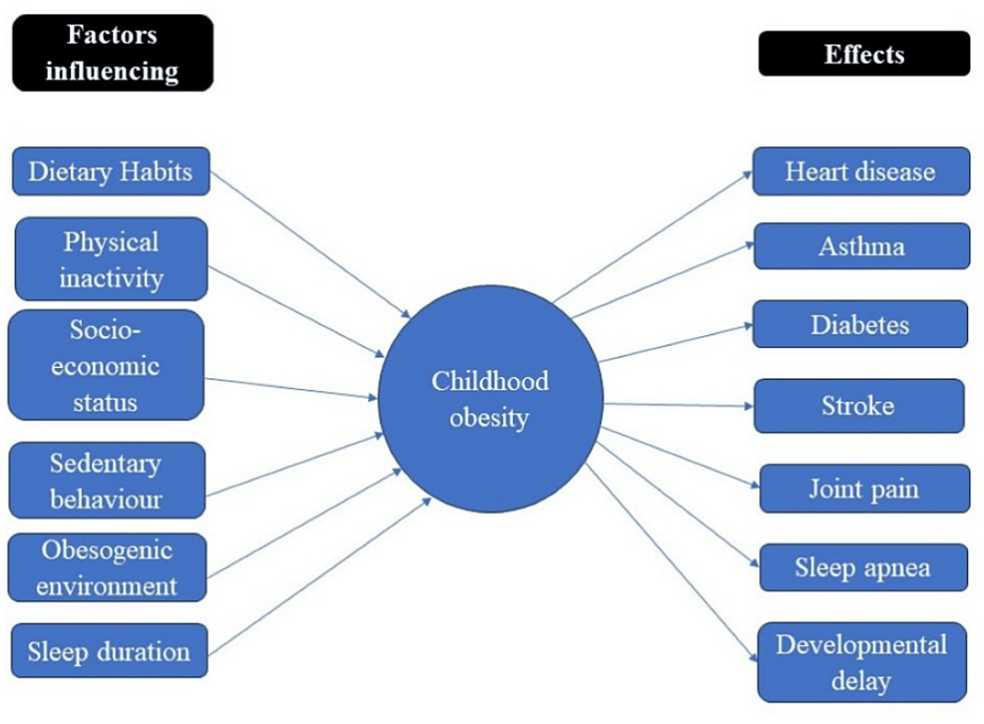
Effects and influencing factors of childhood obesity. Image credits: Sayali Umekar

Childhood obesity is a major public health issue on a global scale. In some poor countries, the prevalence is still low, but it is rising in developed and Western nations. The prevalence of obesity is higher in South America and the eastern Mediterranean (between 30% and 40%) than in Europe (20-30%), Southeast Asia (10-20%), the Western Pacific (10-20%), and Africa (10-20%) [2]. Obesity affects 155 million school-aged children, or one in 10. Recent years have seen a sharp increase in overweight and obesity in many emerging nations, especially in metropolitan areas and among individuals with greater socioeconomic status [2,23]. Obesity was more prevalent in Russian rural areas but less prevalent in Chinese urban areas. Surprisingly, in Russia and China, obesity and overweight rates were observed to be higher among children compared to teenagers [24]. However, in the United States, this pattern did not hold true. Studies conducted in India have revealed that 10-30% of adolescents fall into the category of being overweight or obese [2,24]. Several investigations in India have found that 10-30% of adolescents are fat [2,25]. Figure 2 depicts the factors and effects of childhood obesity.

The most prevalent preventive and therapeutic therapies used and recommended by studies include nutritional, PA, lifestyle, and educational methods [4,26].

Factors Influencing Obesity

Diet: It should be highlighted that calorie restriction is not the only nutritional strategy for combating pediatric obesity; rather, one of the most effective nutrition strategies for achieving sustained weight loss and enhancing both metabolic and mental health involves transitioning to a diet focused on making healthier food choices [4,27]. This diet plan calls for avoiding processed meals with high fat content, fast food, sweetened beverages, refined grains, added sugars, and high-calorie snacks. Instead, it emphasizes the inclusion of fruits, vegetables, nuts, whole grains, and balanced meal timings for a healthier eating pattern [4,28]. Food plays an important role in promoting a healthy diet by not taking food that has high sugar content, reducing intake of salt, and doing regular PAs. Increasing children's and parents' nutritional literacy (in farming, the food sector, security of food cooking, and theoretical understanding of energy balance, dietary habits, and diets) could help to create long-term improvements that contribute to healthy eating habits [4,29]. These are the general principles, but no single diet has been recommended as best for children with obesity [4,27].

PA: The WHO estimates that 20% of the world's adolescent population is physically active enough [30]. The WHO's Global Action Plan on Physical Activity 2018-2030, which was published in June 2018, provides suggested actions that governments can take to encourage PA [31,32]. PA is an important element in the prevention of obesity in the pediatric population. Parents should provide a good example for their children by leading a healthy lifestyle. Prevention programs should include the child's family in particular [30]. Obese children and adolescents face challenges in engaging in PA programs, such as struggles with self-discipline, the absence of a workout partner, and concerns about their appearance leading to self-consciousness [4,33]. Children can engage in sports and other PAs outside of planned fitness courses. Active transportation to school, unstructured active play during school recess, and activities both at home and on playgrounds can serve as legitimate sources of PA [4,34]. After the pandemic, home exercise is highly recommended.

Lifestyle/education: Beyond diet and PA, incorporating other lifestyle factors, such as psychological behaviors and adjusting sleep patterns, is recognized as effective in weight management programs. These supplementary lifestyle adjustments, either independently or in conjunction with educational interventions, have the potential to enhance the effectiveness of PA and dietary interventions. Adjusting sleep patterns in school-age children has shown to positively influence healthy dietary habits by reducing overall food consumption, thereby contributing to favorable weight outcomes [4].

There are actions that should be taken by the government quickly to prevent and treat the childhood obesity targets by 2025, by recommendation of the Commission on Ending Childhood Obesity (Table 1) [31].

**Table 1:** Recommendations of the Commission on Ending Childhood Obesity to meet the government's targets by 2025. Source [31]

Recommendation
Enhance the surroundings where children learn, play, and live to foster holistic development.
Enhance the assessment of dietary and physical activity environments while also refining the implementation of policies to promote healthier lifestyles.
Implement policies that cultivate nourishing eating environments for mothers, infants, and children to support overall well-being.
Amplify policy focus to ensure that children of every age have secure and easily accessible spaces for engaging in physical activities.
Make it your goal to provide everyone with complete health coverage, making sure that kids, teens, and their families can get the resources they need to prevent and manage obesity.

Prevention/Control

**Figure 3:**
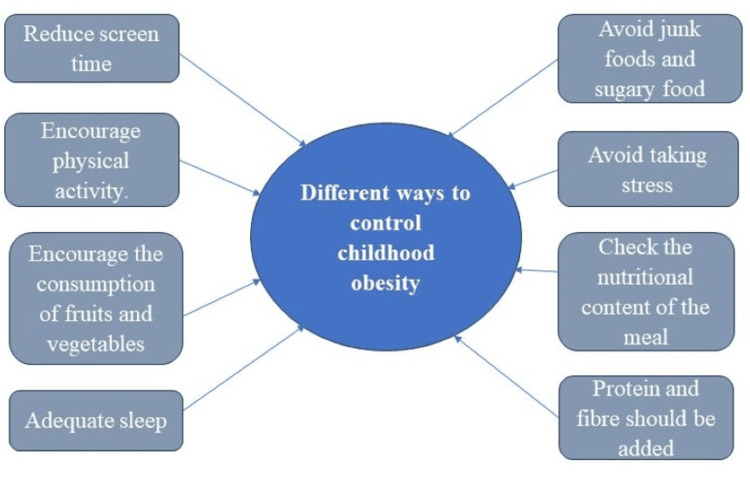
Different ways to control childhood obesity. Image credits: Sayali Umekar

Children living with obesity may benefit from improving their motor abilities to feel better about themselves. It suggests that reducing sedentary activities, family collaboration, and promoting PA in conjunction with dietary education may be critical variables in preventing childhood obesity. Figure 3 depicts different ways to control childhood obesity [2].

Preventive Intervention

Apart from genetic predisposition, there are identified modifiable factors contributing to childhood obesity. Implementing a multifaceted strategy that includes political, social, and educational initiatives is crucial in addressing this issue and targeting various aspects beyond genetics to reduce childhood obesity [35]. Table 2 shows interventions according to child age. From birth to two years, to promote healthier beverage choices among childred, it is advisable to steer clear of sugar-laden options, like flavored milk, including varieties, such as chocolate and strawberry, which often contain added sugars. In two to 12 years, sugary foods, like cakes, biscuits, candy, pastries, and fizzy drinks, are avoided. PAs, like regular exercise, walking, and cycling, should be done every day. Children between 13 and 18 years old should be taught how to plan and prepare meals [35].

**Table 2:** Interventions according to children's age.

Children age	Intervention
Birth–2 years	Encourage exclusive breastfeeding and avoid high-sugar foods. Protein consumption should be under control. Promote the intake of a diverse array of nutritious foods. Avoid eating habits that are restricting. Limit screen time.
2–12 years	Encourage food literacy and education. Avoid sugary food five meals each day. Do 60 minutes of physical activity every day. Limit screen time. Eat vegetables and fruits every day.
13–18 years	Do 60 minutes of exercise. Educate the way to plan and cook meals.

School-based intervention: One of the most crucial settings for preventing childhood obesity is schools, as they have a significant influence on children's education. Schools give education and nourishment and can promote a healthy lifestyle [35]. Education should be given to both parents and children. Promoting a healthy lifestyle requires a multifaceted approach that addresses access to nutritious foods, education on nutrition, creating healthy environments, prioritizing PA, and offering support and guidance for exercise. By focusing on these key components, individuals and communities can work toward achieving and maintaining optimal health and well-being [34].

Family-based intervention: Considering the crucial influence parents have on shaping their children's dietary preferences, PA habits, and sleep patterns, implementing preventative programs centered around families emerges as a practical strategy in addressing the global obesity epidemic. Parents should provide a wide variety of foods, focusing on fruits and vegetables, and restrict the use of sugar-sweetened beverages [35,36]. Encourage educational sessions that highlight the significance of maintaining a healthy weight, adopting an active lifestyle, and avoiding tobacco smoke. Emphasize the importance of a balanced diet, regular PA, and consistent weight monitoring [35].

Community-based intervention: While the significance of both school and family in shaping children's education is undeniable, it is essential to recognize that their educational experiences are also shaped by the broader social environment that surrounds them. As a result, community preventative programs should be promoted, such as taxation of harmful foods and beverages. In urban areas, an open free space for exercise should be available. By combining economic incentives, health initiatives, counseling services, and educational programs, which create a holistic approach that addresses the multifaceted challenges faced by rural communities and low-income families [35].

## Conclusions

The review highlights the critical need for early intervention and prevention strategies to curb the rising prevalence of childhood obesity. These efforts should promote healthier lifestyles, encourage PA, and foster nutritional education at home and in schools. In addition, the role of policy changes, such as improved access to nutritious foods and restrictions on marketing unhealthy products to children, cannot be overstated. Collaborative efforts between healthcare providers, educators, and families are essential to create a supportive environment for children to adopt and maintain healthier habits.
